# Epicardial left atrial appendage occlusion with a new medical device: assessment of procedural feasibility, safety and efficacy in a large animal model

**DOI:** 10.1186/s13019-020-01096-0

**Published:** 2020-04-03

**Authors:** Maximilian Y. Emmert, Michael S. Firstenberg, Arthur T. Martella, Liming Lau, Stephen Zlock, Ashik Mohan, Taylor Spangler, Sarah Currie, Sacha P. Salzberg, Etem Caliskan

**Affiliations:** 1 Department of Cardiovascular Surgery, Charité Universitätsmedizin Berlin, Berlin, Germany; 2 Department of Cardiothoracic and Vascular Surgery, German Heart Center Berlin, Berlin, Germany; 3 Department of Surgery, Summa Akron City Hospital, Akron, USA; 4 Our Lady of Lourdes Medical Center, Camden, USA; 5 Getinge Datascope Corp, Fairfield, USA; 6 Preclinical Medevice Innovations, San Carlos, USA; 7 Heart Rhythm Center Zurich, Zurich, Switzerland

**Keywords:** Atrial fibrillation, Stroke, Oral anticoagulation, Left atrial appendage occlusion, Warfarin, Epicardial

## Abstract

**Background:**

Left atrial appendage occlusion (LAAO) represents a treatment alternative to anticoagulation in patients with atrial fibrillation. We evaluate a novel device for epicardial LAAO in a translational canine model.

**Methods:**

Nine hounds (*n* = 9) were used to assess usability, safety, and efficacy of the TigerPaw Pro (TPP) device for epicardial LAAO. Following baseline imaging (intra-cardiac echocardiography (ICE) and angiography) and intraoperative visual inspection, usability was tested via a ``closure/re-opening`` maneuver followed by deployment of a total of twenty TPP devices (*n* = 20) on the left and right atrial appendages respectively. Procedural safety was evaluated by assessing for adverse-events via direct Epicardial inspection and endocardial imaging. Efficacy evaluation included assessment of device positioning, presence of residual stumps and completeness of closure. Post-mortem evaluation was performed to confirm safety and efficacy.

**Results:**

Usability testing of all TPP devices was successful (*n* = 20;100%, delivery-time range 22–120 s) without any procedural adverse-events (tissue damage or tears, bleeding, vessel-impingement, structural impact). All devices fully traversed the ostium (*n* = 18) or appendage body (*n* = 2), and conformed smoothly to adjacent cardiac anatomy. In nineteen deployments (*n* = 19;95%), all device connector pairs were fully engaged, while in one TPP device the most distal pair remained unengaged. ICE and post-mortem inspections revealed complete closure of all appendage ostia (*n* = 18;100%) and only in one case a small residual stump was detected. Intraoperative safety findings were further confirmed post-mortem. Devices created a nearly smooth line of closure via symmetric endocardial tissue-coaptation.

**Conclusions:**

In this preclinical model, the TPP demonstrated good ease of use for ostial access, ability to re-position (after engagement) and rapid deployment, while achieving safe and effective LAAO.

## Background

Atrial fibrillation (AF) is an independent risk factor for stroke and systemic embolism [1]. Randomized controlled trials (RCTs) have shown oral anticoagulation (OAC) to be effective in the prevention of stroke [2]. As such, vitamin K antagonists (VKAs) have remained the gold-standard treatment for decades. Newer non-vitamin K dependent oral anticoagulants (NOACs) with improved safety and efficacy profiles have lately replaced VKAs as the first-line treatment option [3]. However, the risk of serious bleeding events inherent for all antithrombotic agents requires the development of alternative stroke prevention strategies for patients with AF.

Mechanical exclusion of the LAA, the site of predilection, has been suggested and is currently under investigation [4]. Various surgical exclusion techniques have evolved over the decades. Device-enabled techniques (e.g. surgical stapler devices) replenished these developments; however, none of these achieved reliable and complete closure of the LAA and data on these techniques were mostly inconclusive in their results [4]. The development of percutaneous catheter-based techniques emerged as another promising alternative strategy [5, 6], with robust clinical data. However, due to several limitations (e.g. presence of device-related thrombi, periprocedural complications and residual LAA peri-device leaks) with still-unknown clinical implications, the need for alternative surgical approaches with compelling clinical evidence still exists [4]. The development of new epicardial surgical devices with promising initial clinical results was intended to address this void. First, the AtriClip (AtriCure, Inc.) LAA exclusion system was introduced in 2007 and Initial experience showed an excellent safety, efficacy and durability profile [7] which was further confirmed by imaging-controlled mid- and long-term follow-up results [8, 9].

Another device, the TigerPaw System II (LAAx, Inc., Livermore, CA, now Getinge AB, Sweden) —consisting of an implantable LAA tissue fastener and accompanying delivery tool — was introduced into clinical practice in 2011 following a clinical trial in 2009 [10]. Results from this prospective, multicenter trial [10] demonstrated favorable outcomes - comparable to AtriClip - of this LAA exclusion device. However, despite these encouraging initial data for the TigerPaw System II, the device underwent an FDA Class I recall voluntarily initiated by Maquet, Getinge Group in 2015 due to increased field complaints for incomplete closure of the TigerPaw System II Fastener that may result in tissue tears and/or bleeding [11] . In order to address the intra-procedural issues and to reintroduce the device into clinical use, TigerPaw has undergone systemic re-engineering and redevelopment into a next-generation design, the TigerPaw Pro (TPP) device. In this study, the redesigned TPP for epicardial LAAO was evaluated for procedural feasibility, safety, success and usability in a translational canine model.

## Methods

### Study design and animals

This is the first preclinical study to evaluate the novel TigerPaw Pro (TPP) device for epicardial LAA closure in an in vivo*,* open chest, beating-heart hound model. All procedures were conducted at Preclinical Medevice Innovations (San Carlos, CA, USA), a USDA−/NIH−/FDA-registered and AAALAC-accredited facility. The study was approved by the test facility Institutional Animal Care and Use Committee (IACUC ANS 2156, non-GLP; IACUC ANS 2312, GLP), and was conducted per their standard operating procedures. Overall, nine (*n* = 9) hounds were used. Three animals were used for training the study personnel. Procedures on the other six animals were conducted according to the US FDA 21 C.F.R. §58 Good Laboratory Practice for Nonclinical Laboratory Studies (GLP). The non-GLP training procedures (*n* = 3) and GLP procedures (*n* = 6) were assessed in an identical manner, hence, data from all animals were pooled and presented together.

### TigerPaw pro (TPP) device

The TPP device is a mechanical system for surgical occlusion of the LAA during open heart procedures under direct visualization. TPP consists of a flexible implantable tissue clamp (fastener) that is pre-loaded onto a disposable delivery instrument (delivery tool) for deployment at the LAA ostium. The fastener is based on the principle of pledgeted interrupted mattress sutures, using linearly spaced opposing connector pairs to close the LAA ostium. Each connector pair consists of a male pin that, when deployed via the delivery tool, pierces through tissue to lock into the opposing female segment. Connector engagement is permanent, for secure, durable closure of the LAA ostium, and to mitigate the risk of implant migration. The rigid connector pairs are enclosed within and joined together by a soft, compliant silicone housing, which forms an atraumatic exterior to the fastener, to minimize the risk of abrasion or erosion of adjacent tissues. In addition, the soft silicone housing between connector pairs allows the fastener to flex and conform to the morphology of the appendage ostium and adjacent atrium. Following the market withdrawal of TigerPaw System II, the delivery tool was substantially re-engineered to address the intra-procedural performance issues that led to the recall: first, he device jaws can now be reopened and repositioned prior to initiating fastener deployment and the reliability of the fastener deployment mechanism was greatly improved. In brief, the new fastener design replaces the sharp point barbs with blunt bars that have a definitive trial stop location. This design is based on friable tissue suture needles thereby reducing the likelihood of tissue tear or bleeding. Additional design changes that reduce the likelihood for tearing include the single handle design and yellow trigger mechanism, which aid in preventing movement of the deployment mechanism when placing the fastener. Next, small changes to the design, such as changing plastic components to metal, increase the robustness of the mechanism to place the fastener without movement. Taken together, the overall design was refined to reduce the risk of tissue tears or inadvertent puncture, while the delivery tool was redesigned to improve ergonomics.

**Fig. 1:**
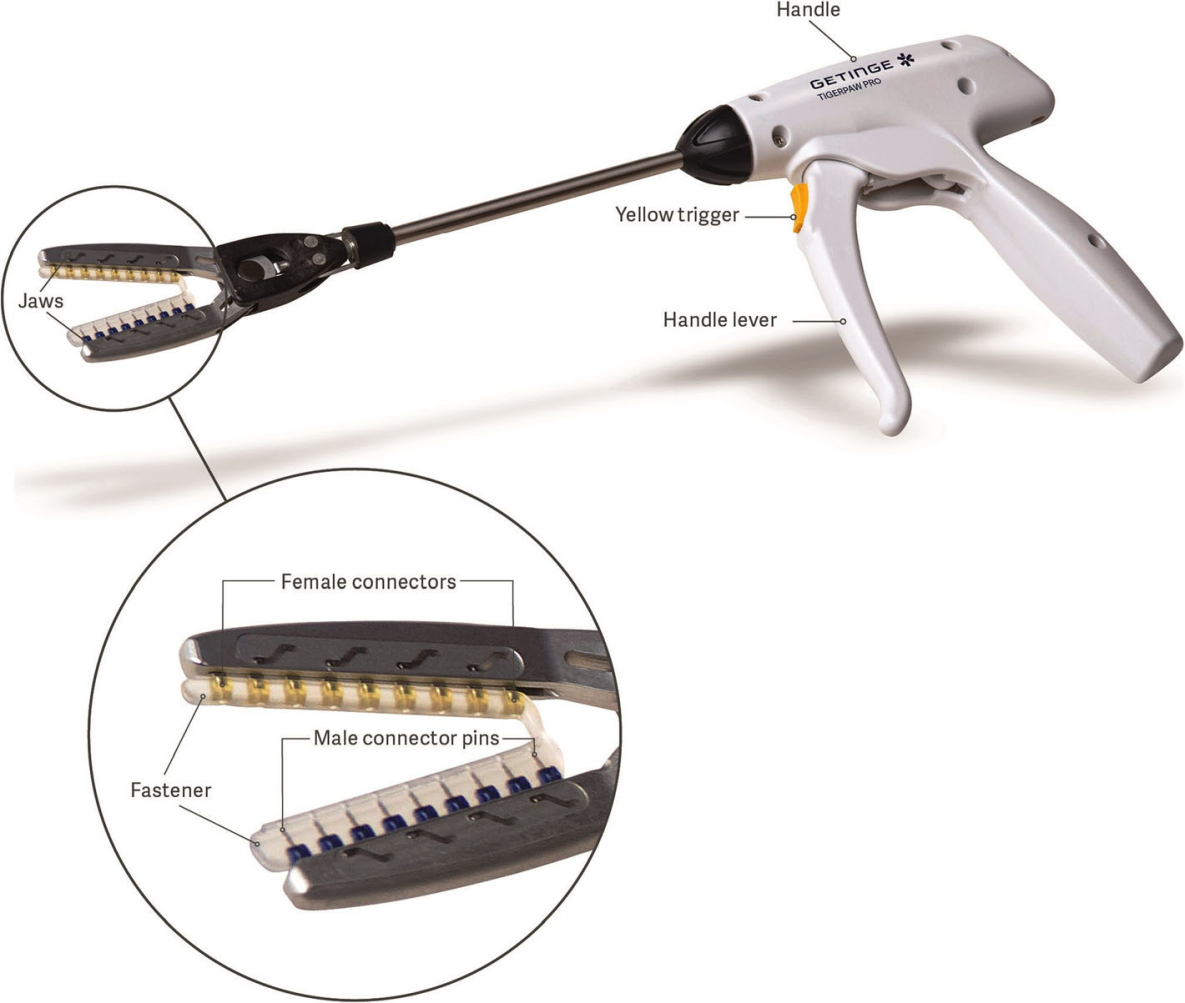
TigerPaw Pro delivery instrument (delivery tool) and flexible implantable tissue clamp (fastener). The fastener is supplied pre-loaded onto the delivery tool jaws as shown. Inset image shows a magnified view of the 45 mm working length fastener, consisting of nine rigid opposing male-female connector pairs enclosed and joined by a soft, compliant housing

However, the fastener implant is largely unchanged from the previous generation, which had an unremarkable safety history post-implantation, and thus is not the focus of this study. The device has two sizes of fastener: 35 mm and 45 mm working length (with seven and nine connector pairs respectively) (Fig. 1).

### Test devices

A total of twenty-one devices (45 mm;*n* = 14 and 35 mm; *n* = 7) were used to conduct the in vivo evaluations **(**Table 1**)**. Of these, twenty devices (*n* = 20) were deployed, while one TPP device (35 mm) was not deployed and intra-operatively exchanged with a larger device (45 mm). All test devices consisted of pre-commercial units, but were representative of the commercial TPP design, manufacture, assembly, and processing.

**Table 1:** Number of TPP fasteners deployed

Location of deployment	Number of fasteners deployed	
	45 mm	35 mm
LAA ostium/neck	9	
RAA ostium/neck	5	4^a^
LAA body	–	1
RAA body		1

^a^One additional 35 mm device was not deployed and intra-operatively replaced with 45 mm device after initial positioning

### Anesthesia, preoperative procedures, surgical access and baseline imaging

The canine model was selected due to its similarities to humans with respect to cardiac-size and morphology. Furthermore, the narrow, deep thoracic cavity of the model can present a challenging geometry for access and device placement, to enhance the rigor of the evaluation. Animal ages ranged from 6.5-11 months (18.6–30.7 kg). Prior to the study, all animals were assessed to be suitable, with no abnormalities noted during a veterinary examination.

Study animals were prepared per standard veterinary procedures for cardiac surgery. Following intubation and start of anesthesia, animals were placed in dorsal recumbency and intermittent positive pressure ventilation was initiated. Monitoring was established for blood pressure, temperature, heart rate, respiratory rate, airway gases, body position, percent oxygen saturation, electrocardiogram, and activated clotting time (ACT). Animals were administered Ringer’s lactate solution via intravenous drip. Lidocaine and/or phenylephrine were administered as needed for visceral analgesia and hypotension. Blood was drawn for complete blood count and serum chemistry.

After assessment of the activated clotting time (ACT), a midline incision was made from the region of the thoracic inlet to the caudal aspect of the rib cage. A sternal saw (Stryker, Kalamazoo, MI, USA) was used to open the thoracic cavity. The pericardium was opened to expose the heart, the right atrial appendage (RAA), and the LAA; next, the pericardium was sutured to the body wall to create a “pericardial cradle”. Following sternotomy procedures, heparin was administered at a dosage determined by the anesthesiologist to maintain an ACT of ≥250 s. ACT (Accriva Diagnostics, San Diego, CA, USA) was monitored every 15-30 min thereafter.

Baseline imagery was obtained using intra-cardiac echocardiography (ICE) (Vivid iq console (GE Healthcare, Chicago, IL, USA); Acunav catheter (Siemens Medical Solutions, Mountain View, CA, USA) and angiography (GE OEC 9800 Plus C-Arm and workstation (GE Healthcare); Cordis diagnostic catheter (Cardinal Health, Dublin, OH, USA); guidewire (Merit Medical Systems, South Jordan, UT, USA); OptiRay contrast (Guerbet, Bloomington, IN, USA) to assess cardiac morphology including LA and LAA ostium, mitral-valve function, left-ventricular function, and the left coronary-system including the left anterior descending (LAD) and circumflex (CX) arteries.

### Device assessment

#### Epicardial evaluation and device testing protocol

The LAA was examined to determine the suitability for TPP application, before it was prepared per the manufacturer’s instructions. Before entering the thoracic space, ACT of ≥250 s was confirmed (for enhanced ability to detect bleeding in the event of device-related tissue injury). The LAA appendage was accessed with the delivery tool (with the pre-loaded fastener) and jaw orientation was adjusted using the rotation knob as needed for an optimal access angle. The device was positioned with the LAA seated as close as possible to the proximal end/elbow of the fastener. Feasibility and usability of a *``closure and re-opening``* maneuver was tested by clamping (closing) the delivery tool jaws on the LAA tissue without initiating fastener deployment, then reopening the jaws (with attached fastener) and removing them from the LAA ostium. Immediately after removal, the site of closure was carefully inspected for potential tissue impact or injury (e.g., tissue damage, tears, bleeding). The inspection was repeated ≥5 min after jaw removal, in case of late or delated adverse-events. All jaw closures for this maneuvers were located on the body of the LAA, away from the neck/ostium so as not to confound the subsequent fastener deployment test.

Next, device deployment was assessed including a) delivery tool jaw positioning, b) jaw closure, c) activation of fastener delivery sequence (to engage connector pairs), and d) release of the fastener from the jaws.

The delivery tool was positioned at the LAA ostium, seating the appendage as close as possible to the proximal elbow of the fastener. The delivery tool jaws were clamped/closed, and visibility and positioning of delivery tool jaws and fastener at LAA target were assessed (i.e., ability to entirely see jaws and fastener, fastener positioned at desired location). At the operator’s discretion, the jaws were reopened as needed to obtain suitable positioning on the LAA neck/ostium. Jaw placement was assessed to determine if fastener length was sufficient to fully traverse the ostium. If sufficient, the fastener was deployed on the LAA ostium (45 mm, *n* = 9), and the delivery tool was removed from the ostium. Duration of the positioning and deployment sequence was recorded.

### Contralateral and additional applications

The jaw closure and fastener deployment evaluations were repeated on the RAA (*n* = 9), to obtain additional data regarding device use. After intraoperative visual evaluation device size (fastener working length) was selected by the operator for each individual application (45 mm, *n* = 5; 35 mm, *n* = 4).

At the operator’s discretion, additional devices were deployed on the body either of LAA (*n* = 1) or the RAA (n = 1) using a rapid deployment maneuver consisting of the full device actuation sequence uninterrupted by study protocol in-process assessments, to further evaluate device safety and usability. The previously delivered devices were left in place during these maneuvers.

### Post-deployment epicardial and endocardial evaluation

Immediately after fastener deployment and delivery tool removal, the site of fastener deployment and adjacent structures were visually inspected for any signs of injury or adverse-events (e.g., tissue damage, bleeding, hematoma, visible vessel impingement or other structural impact). The assessment was repeated ≥5 min after delivery tool removal, in case of late or delated adverse-events.

In addition to visual inspection for any signs of injury or adverse events (e.g., tissue damage, bleeding, hematoma, visible vessel impingement or other structural impact), fastener deployment on the appendage was systematically assessed with respect to the following characteristics:

1. Ability to place (deploy) fastener at appendage ostium.
2. Completeness of fastener deployment with all connector pairs fully engaged (35 mm: seven connectors; 45 mm: nine connectors).
3. Ability of fastener to conform to local anatomy (geometry of adjacent tissues).
4. Sufficient length to traverse appendage ostium for complete closure.

After deployment, ICE and angiography of the LAA and adjacent structures were repeated to assess: 1.) Device safety: by evaluation for evidence of device related injury or adverse impact (e.g., vessel impingement; structural impact); and 2.) Device efficacy: by evaluation of fastener positioning at the ostium (e.g., remaining stump) and of completeness of closure (i.e., endocardial fluid communication between atrium and appendage).

### Euthanasia and post-mortem evaluation

Upon completion of the in vivo assessment procedures, animals were administered a heparin bolus to mitigate the occurrence of post-mortem thrombus artifact. Intravenous potassium chloride was then administered; cardiac arrest was confirmed prior to conducting necropsy.

Post-mortem evaluation was conducted by a board-certified veterinary pathologist (T.S.) in conjunction with the operators, to assess device safety and performance. First, a brief examination of body systems was performed; then, the cardiovascular system was examined in detail.

### Epicardial examination

Epicardial assessments of the appendages and adjacent structures were repeated, evaluating for the following aspects:

1. Evidence of clinically-relevant device-related injury or adverse impact to LAA or adjacent structures (e.g., tissue damage, bleeding, hematoma, visible vessel impingement or other structural impact).
2. Ability of fastener to be placed (deployed) at the appendage ostium.
3. Ability of fastener to function across a range of appendage tissue thicknesses (all fastener connector pairs fully engaged).
4. Ability of fastener to conform to local anatomy/tissue.
5. Sufficient fastener length to fully traverse appendage ostium.

### Tissue thickness estimates

Thereafter, tissue thickness was approximated using Vernier calipers (Mitutoyo, Aurora, IL) at the initial location of jaw closure without fastener deployment as well as immediately adjacent to the deployed ostial fastener (on both heart/atrial side and appendage side).

### Endocardial examination

Finally, the endocardial surfaces of the atrium and the appendage were assessed for:

1. Device safety: by evaluation for evidence of clinically-relevant adverse device-related impact on endocardial surface at the site of exclusion or at adjacent tissues (e.g., tissue damage, bleeding, hematoma, vessel impingement, thrombus formation, structural impact); and
2. Device efficacy: by evaluation for evidence of endocardial fluid communication between atrium and appendage (i.e., incomplete closure).

## Results

### Baseline pre-deployment imaging & Epicardial evaluation

**Fig. 2:**
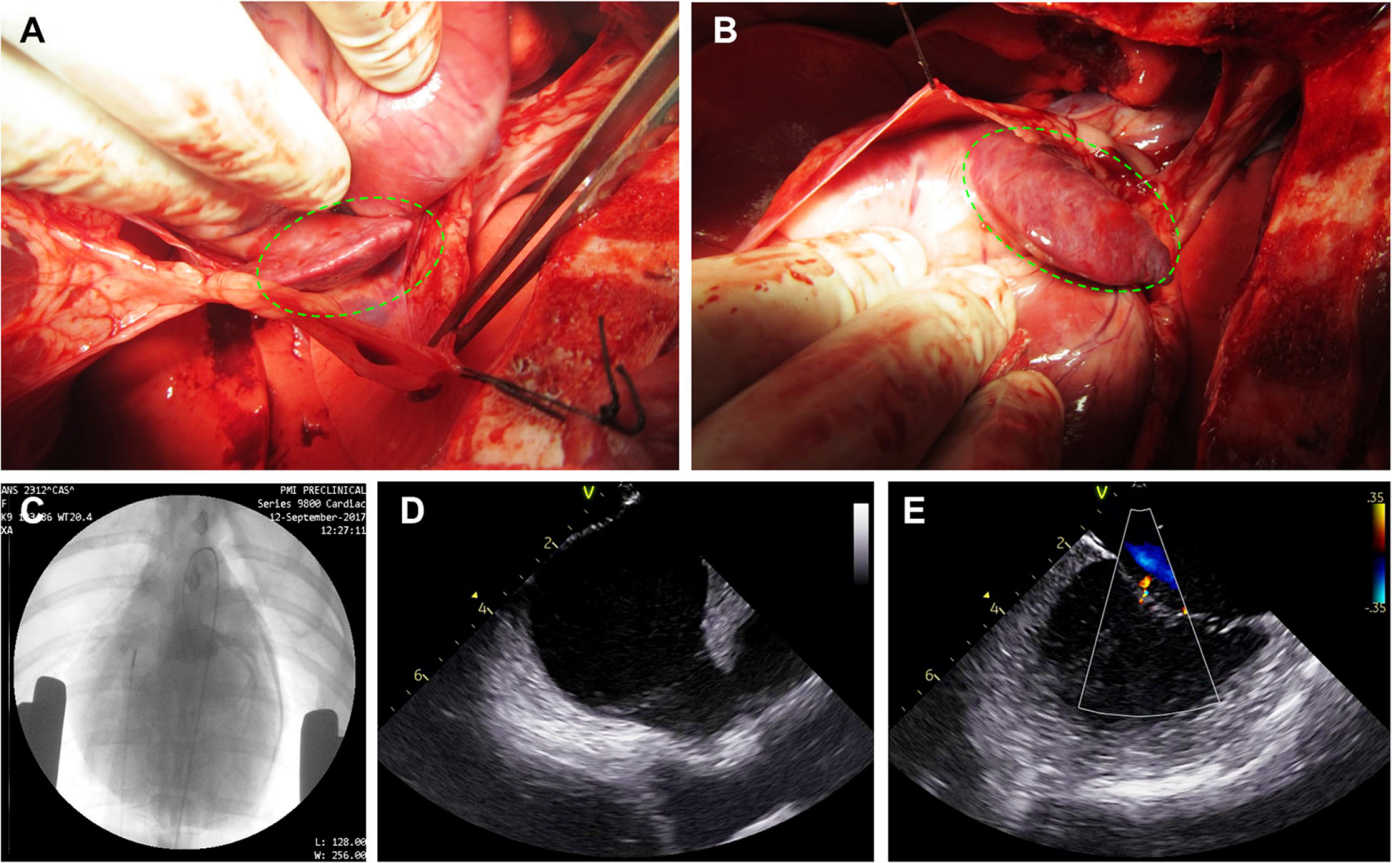
**a**-**e** Epicardial inspection and baseline (pre-deployment) imaging. Visual inspection of LAA and RAA (**a** and **b**). Left coronary angiography (**c**). Intra-cardiac echocardiography of LA and LAA (**d**) and MV (**e**)

All animals displayed a normal cardiac morphology and left-ventricular ejection-fraction. The mitral valve (MV) showed a good function with no (*n* = 2), trace (*n* = 4) or mild (*n* = 3) regurgitation and angiography revealed a normal left-coronary system with a patent LAD and CX in all animals. Epicardial inspection of the appendages showed normal appearance and suitability for deployment in all animals (Fig. 2a-e).

### TPP device testing: appendage clamping and fastener deployment

**Fig. 3:**
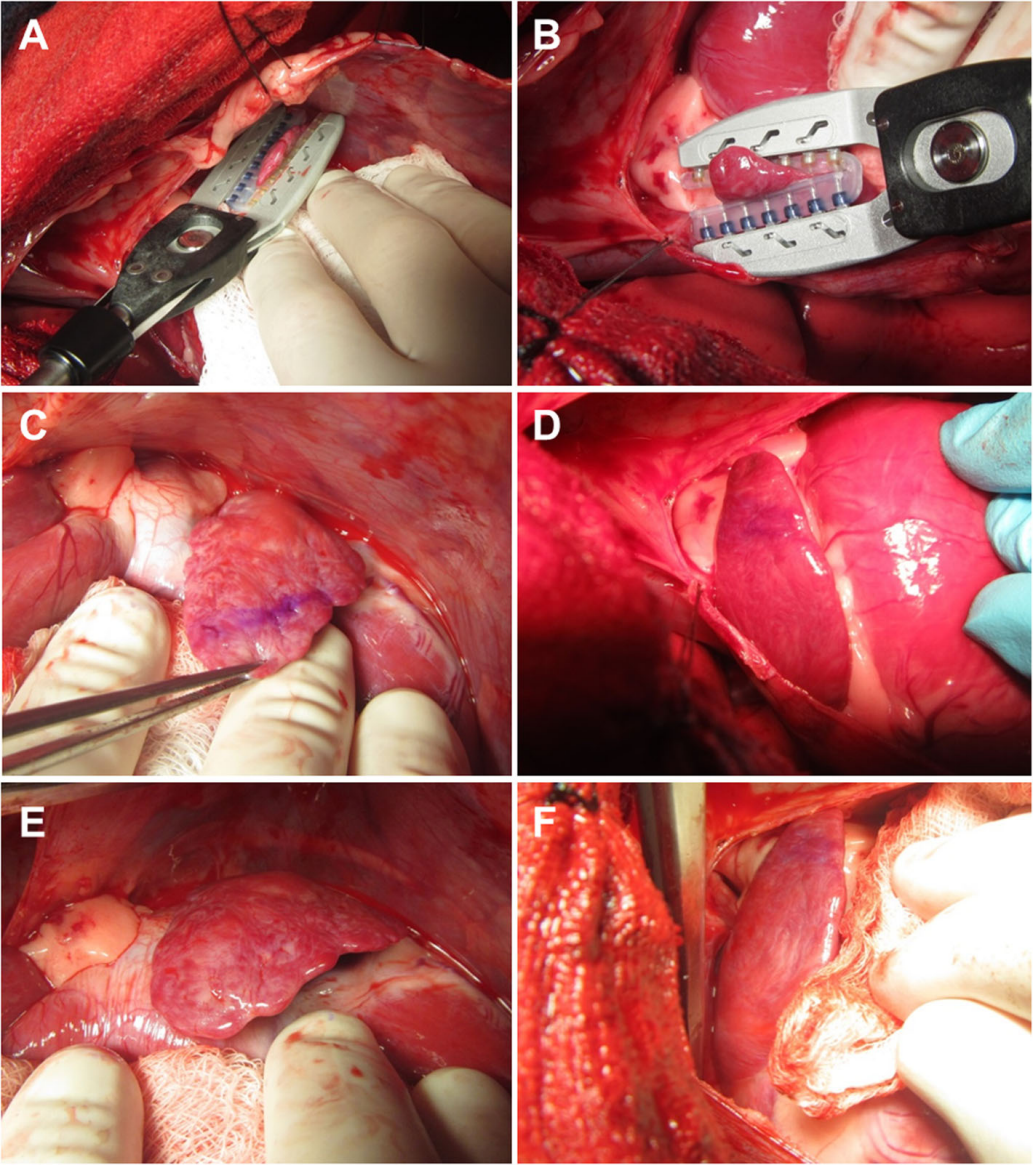
**a**-**f** Appendage access with delivery tool and fastener safety assessment. Jaw closure on LAA (left) and RAA (right) (**a** and **b**). Safety assessment of LAA (left) and RAA (right) immediately (**c** and **d**) and ≥ 5 min (**e** and **f**) after jaw reopening and removal. Location of jaw closure is marked with purple surgical ink

**Fig. 4:**
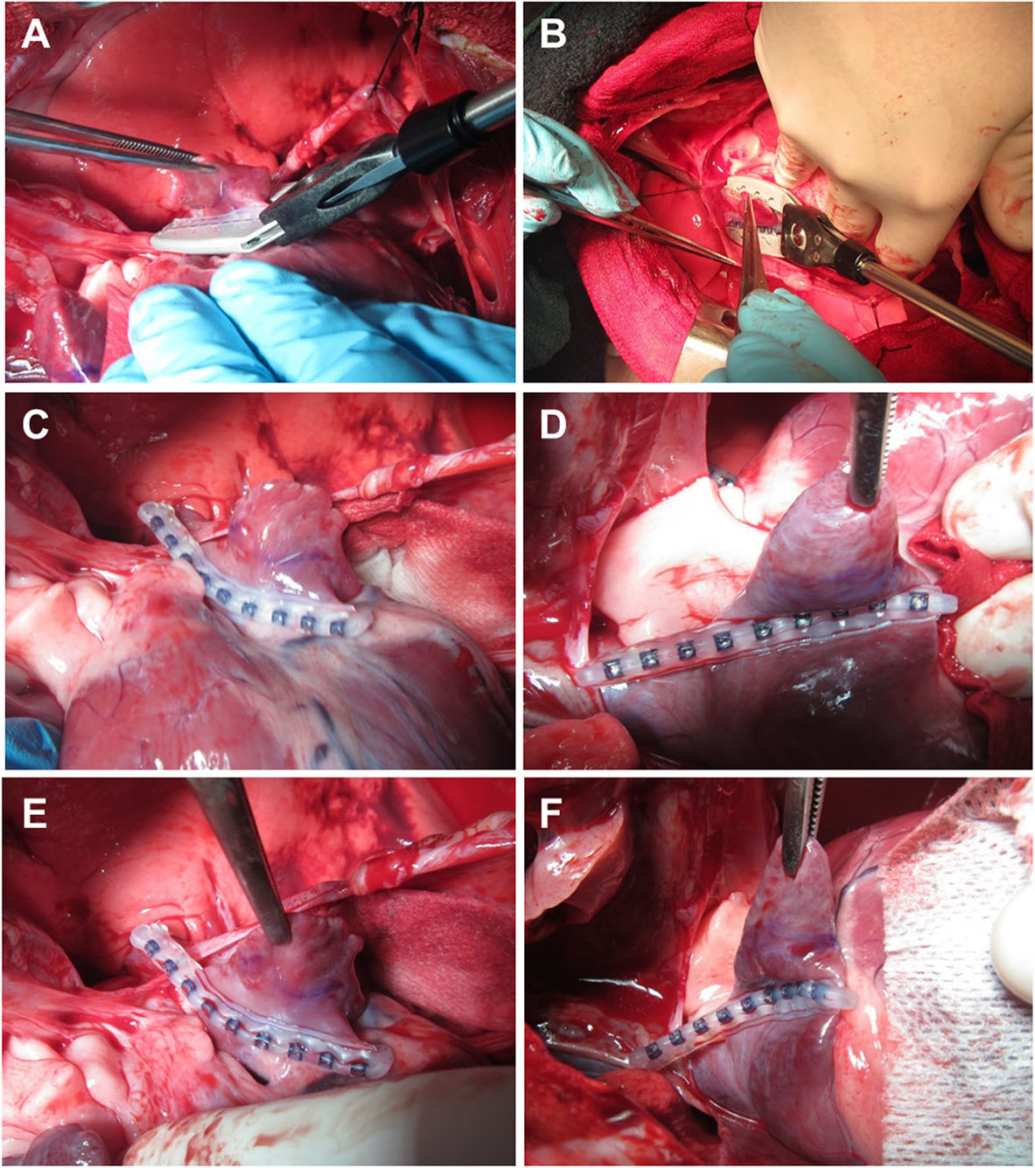
**a**-**f** Delivery tool positioning, deployment, removal, and post-deployment epicardial assessment. Jaw closure on LAA ostium/neck (left) and RAA ostium/neck (right), in preparation for fastener deployment (**a** and **b**). Post-deployment assessment of LAA (left) and RAA (right) immediately (**c** and **d**) and ≥ 5 min (**e** and **f**) after fastener deployment

In all nine animals, both the LAA (*n* = 9) and the RAA (n = 9) could be safely accessed and a *``closure and re-opening`` maneuver* was performed uneventfully. No tissue injury was detectable at the site of jaw closure either immediately or 5 minutes after jaw removal (Fig. 3a-f). In one animal, further inspection of the RAA revealed a minimal hematoma on the RAA tip (i.e., not at the site of closure) which was not considered to be device-related, but was most likely caused by surgical forceps during grasping maneuvers to expose the RAA. Deployment of the fastener was uneventful in all animals with delivery of twenty devices. Eighteen devices were delivered to the LAA ostium and to the RAA ostium (Fig. 4a-f). To evaluate the safety and usability of a rapid deployment maneuver (i.e., uninterrupted device actuation sequence), two additional fasteners were deployed onto the body of the LAA and RAA.

No procedural problems or complications such as bleeding, tearing or device migration were observed. Overall duration of ostial deployments, ranged from 22 to 120 s.

In all ostial deployments (*n* = 18) the operators were able to entirely inspect the position of the delivery tool jaws and fastener with respect to the LAA or RAA neck/ostium, and to evaluate the size of the fastener for the ostium (i.e., sufficient fastener length to fully traverse the ostium). Based on this intra-procedural assessment, in four applications, the operator decided to reopen (i.e., unclamp) the delivery tool jaws in order to better position the jaws on the ostium. In one application, after positioning and jaw closure of a 35 mm fastener device on a RAA, the operator removed and exchanged the device with a longer 45 mm fastener device prior to deploying the fastener at the ostium. In two other deployments, a slight lateral movement was necessary to fully release the fastener from the delivery tool jaw. All of these maneuvers could be performed quickly and uneventfully, demonstrating good ease of use of the device.

### Post-deployment evaluations of device safety and efficacy

Post-procedural epicardial assessment confirmed successful deployment of all fasteners (*n* = 20;100%) onto the LAA ostium (*n* = 9), RAA ostium (n = 9), and the LAA (*n* = 1) or RAA body (n = 1) without evidence of structural impact, vessel impingement, bleeding, hematoma or tissue damage. The deployed fasteners curved to conform smoothly to adjacent cardiac-anatomy without compromising any surrounding structures. The fastener length was sufficient to traverse the entire ostium or appendage body in all cases (100%). In nineteen deployments (95%), all fastener connector pairs appeared to be fully engaged, while in one case using the 45 mm fastener, the most distal connector pair of the fastener (ninth of nine pairs) that was positioned beyond the LAA tissue was not engaged. This was attributed by the operator to unintended capture of pericardial tissue within the jaws (fastener tips) temporarily during the positioning step of the deployment procedure, a situation likely induced by the use of the larger 45 mm device (i.e., oversized relative to the target appendage) in the small canine chest. Nevertheless, the device was able to be positioned and deployed at the ostium, and LAAO was complete.

**Fig. 5:**
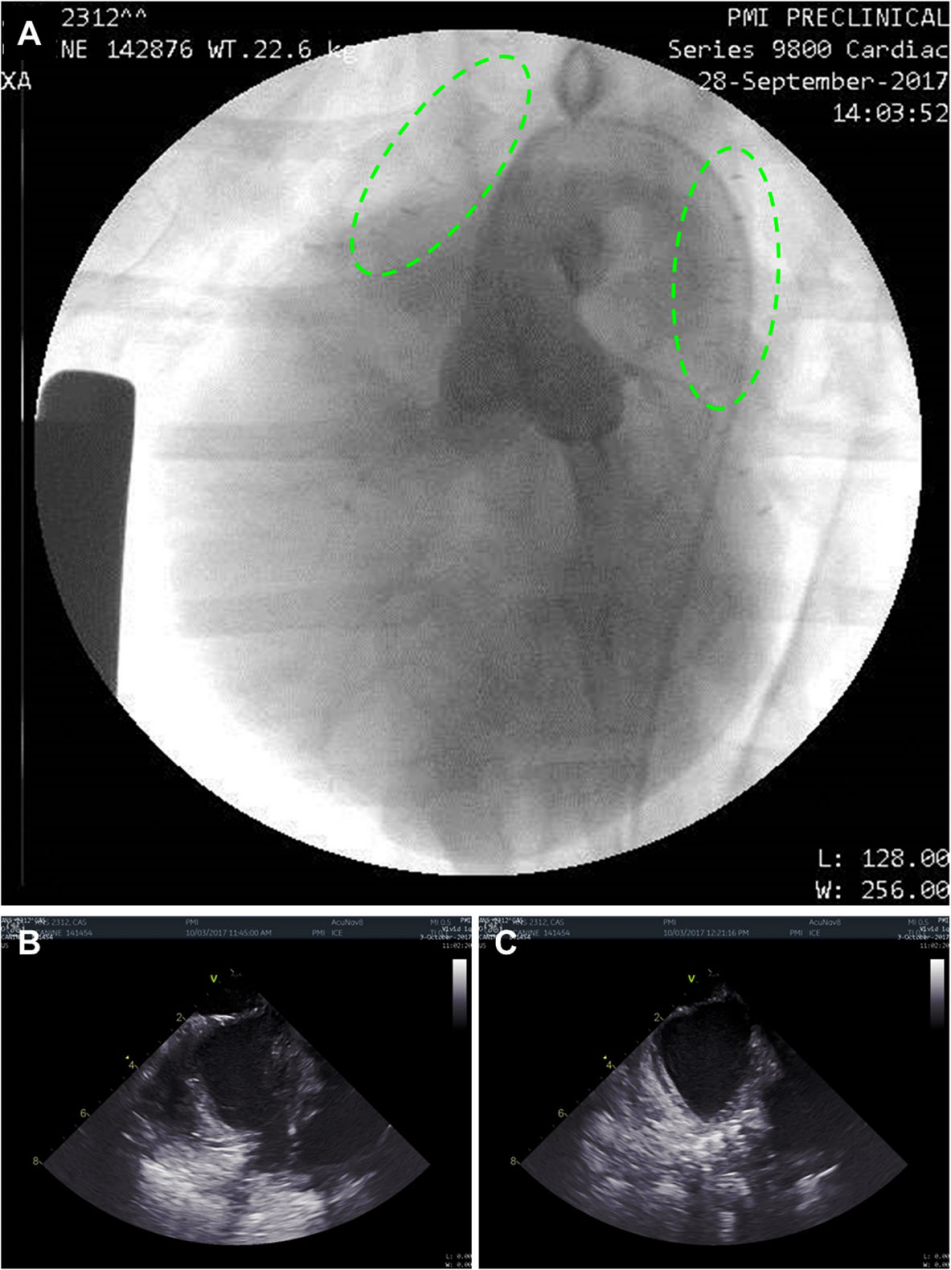
**a**-**c** Post-deployment endocardial assessment. Left-coronary angiography demonstrating patent coronary arteries with two fasteners deployed on the LAA and RAA (green dotted lines) (**a**). Intra-cardiac echo showing the LA and the open LAA pre-deployment (**b**) and the occluded LAA post-deployment of the fastener (**c**)

No evidence of tissue damage or bleeding was detected on endocardial evaluation. Angiography displayed an unchanged left coronary system. In 8/9 animals, ICE assessment of the LAA showed complete LAAO without any evidence of fluid communication (Fig. 5a-c). Complete LAAO could not be confirmed in the ninth animal, but was subsequently confirmed in necropsy, indicating a good efficacy profile of the device (100%).

In eight applications (89%), no LAA stump was detectable, while in one animal a small residual stump was visible suggesting that the device was not exactly positioned on the ostium. This was attributed by the operator to the space constraints in the small canine chest. In the same animal, the MV presented with moderate regurgitation after fastener deployment (increasing from trace regurgitation pre-deployment). However, ICE displayed that the regurgitation jet was central and that the MV was structurally unremarkable, suggestive that this finding was not related to the deployed fastener. MV function was unchanged in the remaining eight animals, and all nine animals otherwise displayed unchanged morphological and functional conditions.

### Post-mortem assessment and clinical pathology

**Fig. 6:**
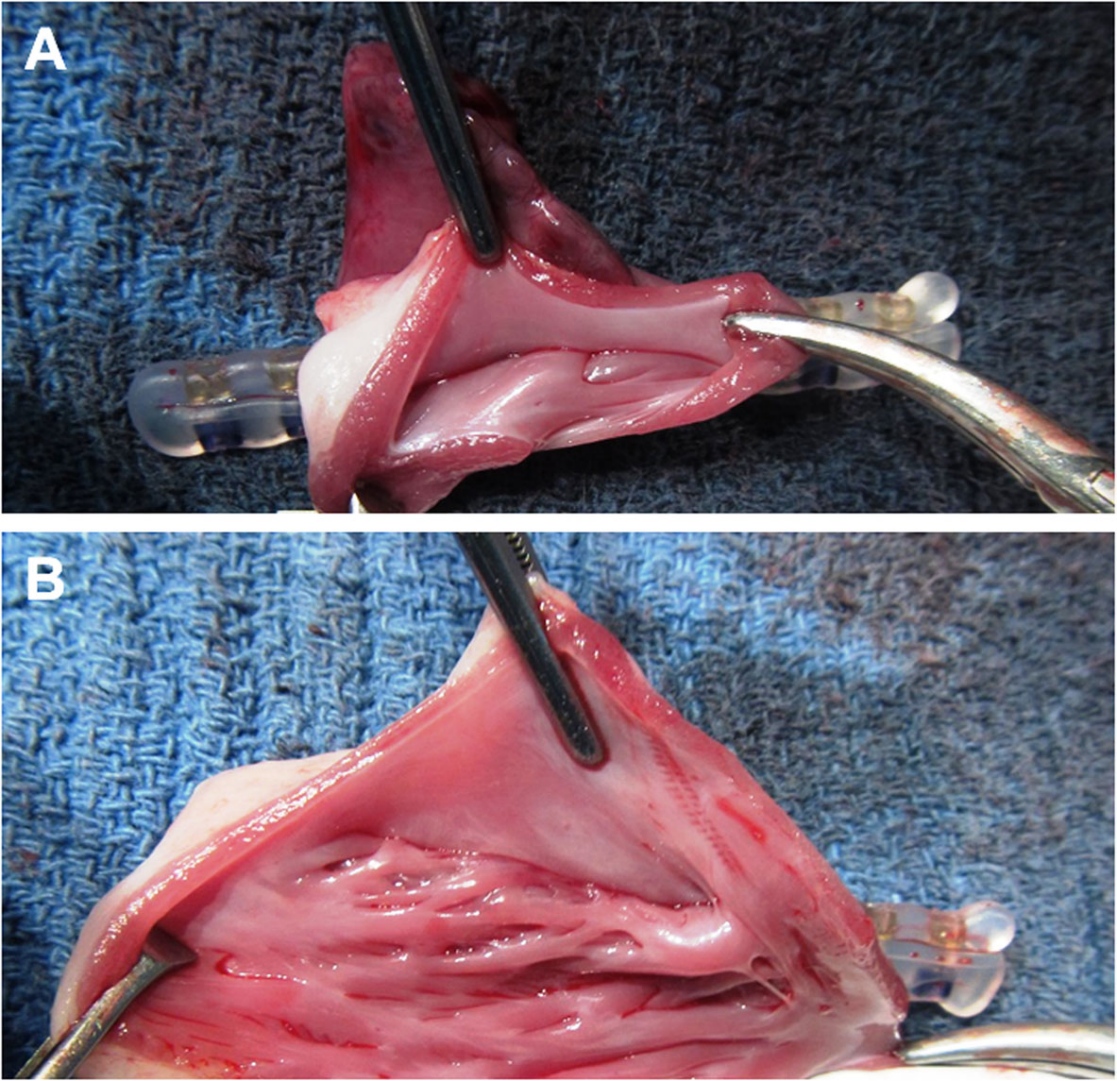
**a**-**b** Post-deployment necropsy assessment. Representative necropsy images of the LAA (**a**) and RAA (**b**) demonstrating a complete LAA closure after faster deployment

Post-mortem epicardial inspection confirmed the intraoperative findings for all animals (*n* = 9). There was no evidence of structural impact, tissue damage, vessel impingement, bleeding or hematoma at the sites of delivery tool jaw clamping/closure (without fastener deployment) (*n* = 18), at the ostial sites of fastener deployment (n = 18), or at the sites of “rapid deployment” on the LAA/RAA body (*n* = 2). For all ostial deployments except one (*n* = 17/18, see above), the device was well positioned at the ostium, conformed to the surrounding cardiac structures and had a sufficient length to fully traverse the appendage (Fig. 6a-b). Consistent with the intraoperative observations, complete engagement of all connector pairs was confirmed in nineteen of twenty deployed fasteners. One of twenty fasteners exhibited incomplete engagement of one connector pair (the most distal one), as previously observed in vivo; complete closure of the LAA was confirmed for this deployment.

Endocardial inspection confirmed for all specimens that there was no communication between the atrium and the appendage, indicating complete closure in all cases. The device-enabled appendage occlusion created a nearly smooth line of closure via symmetric endocardial tissue coaptation. In one animal, two locations of pinpoint congestion were noted on the endocardial surface of the RA adjacent to the deployed fastener; however, this observation was not judged to be related to the fastener but may have eventually occurred during RAA exposure and the various (re-)positioning and re-opening maneuvers. No other evidence of adverse impact was seen at the location of fastener deployment or at adjacent tissue structures.

Tissue thickness measurements indicated that the appendage tissues at the location of delivery tool jaw clamping/closure (without fastener deployment) and immediately adjacent to the deployed fastener at the LAA and RAA ostia ranged from approximately 1.5–7.8 mm and 1.5–6.8 mm on the LAA and RAA respectively.

## Discussion

### TPP device enables safe and efficacious appendage closure

We evaluated the in vivo performance of the new TigerPaw Pro (TPP) device for epicardial LAAO in a translational canine model. Our results demonstrate a good safety and efficacy profile of the device. All deployment procedures could be performed successfully and, importantly, no instances of device-related intra-procedural or post-procedural complications were observed (i.e., bleeding, tearing, other tissue damage, device migration, vessel impingement), substantiating the overall safety of the procedure and suggesting that the issues of the previously recalled TigerPaw System II have been resolved by redesign.

In all cases, complete closure of the appendage was achieved, as validated by ICE, visual inspection and post-mortem evaluation. The examinations showed that a smooth endocardial surface was achieved (via symmetric tissue coaptation) after fastener deployment, which reduces the potential risk of blood-stasis and thrombus formation at the closed ostium, and thus represents an important criterion in appendage occlusion [4].

### TPP device offers good usability, the ability for re-positioning and rapid delivery

The device was extensively tested for its ability to apply the fastener to the appendage (procedural feasibility) and for its intra-procedural ease of use (usability). The device demonstrated a good performance profile, as all testing maneuvers could be safely and precisely performed. In all attempts, after closing and clamping the device jaws on appendage tissue, the jaws could be reopened without issue and without inadvertently initiating fastener deployment. For all fastener applications, access, positioning and deployment at the ostium was safe, rapid, and accurate. In four applications, after clamping tissue but prior to initiating fastener deployment, the operators decided to reopen the jaws to achieve better positioning at the ostium; this maneuver was completed uneventfully, indicating that the device allows for safe and reliable reopening and repositioning, if deemed necessary. Considering the different morphologies the LAA can present with [12], the ability for fast and safe device repositioning represents an important feature for achieving efficient LAAO. Notably, two attempts of a rapid, uninterrupted delivery procedure were successful, providing further evidence of the device’s ability to be deployed quickly.

Two difficulties were encountered during testing; one instance of an unengaged fastener connector pair, and one instance of residual LAA stump. The unengaged connector pair was attributed to unintended capture of pericardial tissue in the jaws during positioning; this situation was likely induced by the use of the larger (45 mm) fastener in the small thoracic space of the canine model. The one instance of LAA residual stump was also related to maneuverability of the larger fastener in the canine chest, and was attributed by the operator to the space constraints of the model. Retrospectively, the smaller (35 mm) device may have avoided both difficulties highlighting the importance of appropriate sizing when targeting the space-limited and fragile appendage environment. Notably, in none of these various maneuvers and device deployments did any technical issues or complications occur, such as tissue damage or tearing, bleeding or device dislodgement, indicating an encouraging usability and safety profile of the device.

### Evidence of epicardial device enabled LAAO

Mechanical occlusion of the LAA (using either surgical or interventional approaches) has been repeatedly suggested as a potential alternative to OAC therapy in patients with AF, and is currently under intense evaluation [4]. To date, only for interventional devices (e.g., Watchman, Boston Scientific, Marlborough, MA, USA) has a reasonable efficacy been demonstrated in prospective randomized clinical trials [6, 13]; the outcomes for surgical techniques still remain heterogeneous, and so far, data from clinical trials remain scarce [4]. In particular, durability and completeness of LAA closure still represent major issues for most surgical approaches, thus limiting the overall therapy efficacy [4]. In this regard, device-enabled epicardial approaches have been repeatedly suggested as a potential option to achieve safe, complete and durable occlusion of the LAA [8]. Notably, 10 years after its introduction and with currently > 200.000 devices sold, the AtriClip device possesses the largest body of clinical experience for device-enabled surgical approaches. Initial results from a prospective European clinical pilot trial and a US-based multicenter trial (EXCLUDE) showed a good safety and efficacy profile for the AtriClip device using systematic imaging in controlled follow-up at 3 months [7, 14]. These data were further validated by computed tomography in controlled mid- and long-term studies demonstrating safe, durable and complete LAA occlusion [9, 15, 16], as well as initial signs of potential reduction of stroke risk in patients with discontinued OAC [8]. A recent systematic review on outcomes of LAA occlusion using the AtriClip device showed the device to be safe and effective in the management of patients with atrial fibrillation, either as an adjunct in patients undergoing cardiac surgery or as a stand-alone thoracoscopic procedure with successful LAA occlusion in 97.8% of patients and no reported device-related adverse events [17].

In 2009, the TigerPaw System (TigerPaw) epicardial LAA occlusion system (LAAx, Inc., Livermore, CA, now Getinge AB, Sweden) was evaluated in a prospective multicenter trial including sixty patients [10], demonstrating short-term outcomes comparable to the AtriClip studies [7, 14]. In all patients, the TigerPaw device could be safely and rapidly implanted (< 30 s). Trans-esophageal echocardiography immediately after delivery and at 3 months follow-up confirmed the absence of any leaks, indicating complete and durable LAA occlusion and further validating the concept of device enabled epicardial LAA closure [10]. However, despite these encouraging initial experiences, the next-generation TigerPaw System II was voluntarily recalled in 2015 due to the increased occurrence of intra-procedural technical problems and related safety issues [11]. In order to address these issues, the TigerPaw II system underwent systematic redesign with a particular focus on improving performance of the delivery tool in the device deployment procedure. Hence, the data presented here are of high relevance as they provide the first in vivo experience with the new TigerPaw Pro design, and may therefore build the basis for further clinical use.

The field of surgical LAA closure remains an important scientific topic, as despite accumulating preliminary evidence [18] and ongoing trial effort (LAAOS III), robust clinical data supporting the efficacy of surgical LAA closure are still scarce. Consequently, current guidelines limit surgical LAA closure to a Class IIb, Level of Evidence B recommendation during open-heart procedures [3]. The experience drawn from this study, along with the experiences from a similar device, the AtriClip, need to be translated into a well-designed clinical trial to provide the long-needed robust evidence for epicardial device-enabled surgical LAA closure. With the still-unknown clinical implications of issues with percutaneous devices, a collaborative effort of both cardiac surgeons and cardiologists in the setting of a Heart Team is of utmost importance, in order to present patients with tailored therapies.

Our study has several limitations: First, this was an acute study, with no long-term follow-up in regard to safety and durability of appendage closure. Our protocol focused on detailed assessment of intra-procedural safety and device usability after its re-design, since these were the primary issues of the previous TigerPaw System II. It must be noted that epicardial LAAO with AtriClip or TigerPaw II have been demonstrated to remain durable during follow-up when complete closure was documented at the time of implantation [4].

Second, all testing was conducted in a preclinical animal model, with a limited number of surgeons and of TPP device used, which necessarily cannot represent the full range of conditions (i.e. appendage size and morphology, type and history of arrhythmia, tissue fragility, etc.) and operator experiences to be expected in actual clinical use. Third, all animals were fully heparinized after sternotomy in order to achieve a coagulation status as in routine cardiac surgery. Due to the acute nature of this study with still active full heparinization at the time of euthanasia, any clot formation along the closure line was unlikely and thus not observed. However, the absence of any bleeding complication or relevant hematoma at the site of application despite full heparinization further supports the safety profile of the device.

## Conclusions

The new TPP device for epicardial LAAO demonstrated safety and efficacy in a preclinical canine model. All deployment procedures were performed safely and successfully, and achieved complete LAAO. The device showed good ease of use with respect to LAA access, the ability for re-positioning (after engagement) and rapid deployment. These data warrant further clinical evaluation.

## Data Availability

The datasets used and/or analyzed during the current study are available on reasonable request.

## References

[1] Wolf PA, Abbott RD, Kannel WB (1991). Atrial fibrillation as an independent risk factor for stroke: the Framingham study. Stroke..

[2] Hart RG, Pearce LA, Aguilar MI (2007). Meta-analysis: antithrombotic therapy to prevent stroke in patients who have Nonvalvular atrial fibrillation. Ann Intern Med.

[3] Kirchhof P, Benussi S, Kotecha D, Ahlsson A, Atar D, Casadei B, et al. 2016 ESC Guidelines for the management of atrial fibrillation developed in collaboration with EACTS. Eur Heart J. 2016;37(38):2893–962. 10.1093/eurheartj/ehw210 27567408

[4] Caliskan E, Cox JL, Holmes DR, Meier B, Lakkireddy DR, Falk V (2017). Interventional and surgical occlusion of the left atrial appendage. Nat Rev Cardiol.

[5] Holmes DR, Doshi SK, Kar S, Price MJ, Sanchez JM, Sievert H (2015). Left atrial appendage closure as an alternative to warfarin for stroke prevention in atrial fibrillation: a patient-level meta-analysis. J Am Coll Cardiol.

[6] Holmes DR, Reddy VY, Turi ZG, Doshi SK, Sievert H, Buchbinder M (2009). Percutaneous closure of the left atrial appendage versus warfarin therapy for prevention of stroke in patients with atrial fibrillation: a randomised non-inferiority trial. Lancet.

[7] Salzberg SP, Plass A, Emmert MY, Desbiolles L, Alkadhi H, Grunenfelder J (2010). Left atrial appendage clip occlusion: early clinical results. J Thorac Cardiovasc Surg.

[8] Caliskan E, Sahin A, Yilmaz M, Seifert B, Hinzpeter R, Alkadhi H (2018). Epicardial left atrial appendage AtriClip occlusion reduces the incidence of stroke in patients with atrial fibrillation undergoing cardiac surgery. Europace.

[9] Emmert MY, Puippe G, Baumuller S, Alkadhi H, Landmesser U, Plass A (2014). Safe, effective and durable epicardial left atrial appendage clip occlusion in patients with atrial fibrillation undergoing cardiac surgery: first long-term results from a prospective device trial. Eur J Cardio-Thoracic Surg.

[10] Slater AD, Tatooles AJ, Coffey A, Pappas PS, Bresticker M, Greason K (2012). Prospective clinical study of a novel left atrial appendage occlusion device. Ann Thorac Surg.

[11] U.S. Food and Drug Administration (2015). Class 1 Device Recall TigerPaw System II.

[12] Wang YAN, Di Biase L, Horton RP, Nguyen T, Morhanty P, Natale A (2010). Left atrial appendage studied by computed tomography to help planning for appendage closure device placement. J Cardiovasc Electrophysiol.

[13] Holmes DR, Kar S, Price MJ, Whisenant B, Sievert H, Doshi SK (2014). Prospective randomized evaluation of the watchman left atrial appendage closure device in patients with atrial fibrillation versus long-term warfarin therapy: the PREVAIL trial. J Am Coll Cardiol.

[14] Ailawadi G, Gerdisch MW, Harvey RL, Hooker RL, Damiano RJ, Salamon T (2011). Exclusion of the left atrial appendage with a novel device: early results of a multicenter trial. J Thorac Cardiovasc Surg.

[15] Kurfirst V, Mokracek A, Canadyova J, Frana R, Zeman P (2017). Epicardial clip occlusion of the left atrial appendage during cardiac surgery provides optimal surgical results and long-term stability. Interact Cardiovasc Thorac Surg.

[16] Caliskan E, Eberhard M, Falk V, Alkadhi H, Emmert MY (2019). Incidence and characteristics of left atrial appendage stumps after device-enabled epicardial closure. Interact Cardiovasc Thorac Surg.

[17] Toale C, Fitzmaurice GJ, Eaton D, Lyne J, Redmond KC (2019). Outcomes of left atrial appendage occlusion using the AtriClip device: a systematic review. Interact Cardiovasc Thorac Surg.

[18] Friedman DJ, Piccini JP, Wang T, Zheng J, Malaisrie SC, Holmes DR (2018). Association between left atrial appendage occlusion and readmission for thromboembolism among patients with atrial fibrillation undergoing concomitant cardiac surgery. JAMA..

